# The Sialidase NanS Enhances Non-TcsL Mediated Cytotoxicity of *Clostridium sordellii*

**DOI:** 10.3390/toxins8060189

**Published:** 2016-06-17

**Authors:** Milena M. Awad, Julie Singleton, Dena Lyras

**Affiliations:** Infection and Immunity Program, Biomedicine Discovery Institute and Department of Microbiology, Monash University, Clayton 3800, Australia; milena.awad@monash.edu (M.M.A.); julie.singleton@monash.edu (J.S.)

**Keywords:** clostridia, toxins, lethal toxin, sialidase, pathogenesis, *Clostridium sordellii*

## Abstract

The clostridia produce an arsenal of toxins to facilitate their survival within the host environment. TcsL is one of two major toxins produced by *Clostridium sordellii*, a human and animal pathogen, and is essential for disease pathogenesis of this bacterium. *C. sordellii* produces many other toxins, but the role that they play in disease is not known, although previous work has suggested that the sialidase enzyme NanS may be involved in the characteristic leukemoid reaction that occurs during severe disease. In this study we investigated the role of NanS in *C*. *sordellii* disease pathogenesis. We constructed a *nanS* mutant and showed that NanS is the only sialidase produced from *C. sordellii* strain ATCC9714 since sialidase activity could not be detected from the *nanS* mutant. Complementation with the wild-type gene restored sialidase production to the *nanS* mutant strain. Cytotoxicity assays using sialidase-enriched culture supernatants applied to gut (Caco2), vaginal (VK2), and cervical cell lines (End1/E6E7 and Ect1/E6E7) showed that NanS was not cytotoxic to these cells. However, the cytotoxic capacity of a toxin-enriched supernatant to the vaginal and cervical cell lines was substantially enhanced in the presence of NanS. TcsL was not the mediator of the observed cytotoxicity since supernatants harvested from a TcsL-deficient strain displayed similar cytotoxicity levels to TcsL-containing supernatants. This study suggests that NanS works synergistically with an unknown toxin or toxins to exacerbate *C. sordellii*-mediated tissue damage in the host.

## 1. Introduction

*Clostridium sordellii* is a Gram positive, spore-forming anaerobe, and an important pathogen of humans and animals that causes a range of severe diseases [1,2,3,4]. The human clinical spectrum of disease includes toxic shock, particularly in postpartum and post abortive women, myonecrosis (gas gangrene) and sepsis [5] and, although such infections are rare, they result in a high mortality rate of 70% to 100%. The characteristic features of *C. sordellii* infection include rapid tissue necrosis, shock, multi-organ failure, and diffuse capillary leak with an exacerbated circulating white blood cell count (leukemoid reaction) [1,6], the latter two being hallmark characteristics of infections caused by this bacterium.

Clostridial diseases are mostly toxin-mediated. *C. sordellii* produces numerous extracellular toxins including lethal toxin (TcsL), the best studied *C. sordellii* toxin, but also haemorrhagic toxin (TcsH), phospholipase C (PLC), sordellilysin (SDL) and sialidase (NanS, a neuraminidase) [7]. Only lethal toxin (TcsL) has been shown to be essential to the pathogenesis of *C. sordellii*-mediated disease [8,9] with few studies examining the role that the other toxins may play in an infected host. In 2007, a study by Aldape *et al.* demonstrated that NanS induced promyelocytic cell proliferation *in vitro*, suggesting that it played a role in the characteristic leukemoid reaction that occurs with severe, and often fatal, *C. sordellii* infections [1].

Sialic acid residues are terminal sugars found on the surface of many eukaryotic cells, often as components of secreted glycoproteins including blood related proteins and mucins [10]. These residues are involved in numerous cellular processes such as masking ligand binding receptors or facilitating ligand binding, cell to cell interactions, cell signalling processes and regulating innate immunity [11,12,13]. Microbial pathogens also produce sialic acids or scavenge host sialic acids. These sialic acids are located on the microbial surface and are used as a subversive tactic to protect against host immune defences [11]. In addition, pathogens utilize host sialic acids as a nutritional energy source [11,14].

Sialidases hydrolyse terminal sialic acid residues from host glycoproteins, glycolipids, and oligosaccharides [15,16] and contribute to the virulence capacity of many microbial pathogens [15,17,18,19]. Sialidase-mediated virulence mechanisms vary and include the enhancement of biofilm formation [20], increased bacterial adherence to host cells [10,16], and augmenting the cytotoxicity of specific toxins [16,17,21,22]. These include NanI from *Clostridium perfringens*, which enhances the cytotoxicity of epsilon toxin (ETX) [16,21] and NANase from *Vibrio cholera*, which appears to enhance the binding and uptake of cholera toxin [22].

Sialidases are ubiquitous in the clostridia and these enzymes have been described in *Clostridium septicum*, *Clostridium chauvoei* and *C. perfringens* [17,23]. The best-studied clostridial sialidases are from *C. perfringens*, which encodes up to three sialidases, NanI, NanH and NanJ [16,17]. NanI and NanJ do not contribute directly to the virulence of *C. perfringens* strain 13 in an *in vivo* mouse infection model but these enzymes enhance the activity of alpha-toxin and perfringolysin O in culture supernatants and alpha toxin in a cell culture model [17]. Enhanced alpha-toxin activity resulting from the action of NanI was also shown in another study, where the cytotoxic effect of alpha-toxin was significantly increased, in a dose-dependent manner, by NanI in an epithelial tumour cell line and in Chinese hamster lung fibroblasts [24]. Furthermore, Li *et al.* reported a similar finding where the effect of epsilon toxin (ETX) on MDCK cells was enhanced by NanI [16].

Mucosal surfaces of the lung, gastrointestinal tract and vagina are heavily glycosylated and sialic-acid rich [15]. Studies published in 2006 showed that *C. sordellii* colonises the gastrointestinal tract of approximately 0.5% of human adults and the vagina of 0.5%–10% of women [5], although recent figures indicate rates of carriage of 3.2% and 0.2% in the gut and vagina, respectively [25]. *C*. *sordellii* encodes at least one sialidase, NanS [1,7,26], which has been hypothesised to play a role in cell adhesion and/or the enhanced cytotoxicity of its various toxins within the sialic acid-containing gastrointestinal tract and vaginal host niches.

In this study, we report the construction and genetic complementation of a *nanS* mutant in the *C. sordellii* type strain ATCC9714. Phenotypic analysis was used to determine the contribution of NanS to *C. sordellii* sialidase activity and to determine if this enzyme enhances the cytotoxic effects of the major toxin, TcsL. Our results show that NanS is secreted and appears to be the sole contributor to sialidase activity in this strain, under the conditions tested. Importantly, although NanS does not appear to enhance TcsL activity, it augments the action of an unknown virulence factor produced by strain ATCC9714 on host cells, since cell cytotoxicity was enhanced in the presence of sialidase.

## 2. Materials and Methods

### 2.1. Bacterial Strains and Culture Conditions

All *C. sordellii* strains used in this study were derivatives of ATCC9714 (Table 1) and were cultured either in Heart infusion broth (HIS) (Oxoid) supplemented with 0.1% l-cysteine HCL and 0.5% glucose or Todd Hewitt (TH) broth (Oxoid) supplemented with 0.02% glucose and 0.005% l-cysteine. All clostridial media were pre-reduced in an anaerobe chamber (Coy) for 24 h prior to inoculation, and cultures or solid media were incubated at 37 °C under anaerobic conditions (10% H_2_, 10% CO_2_ and 80% N_2_) for 24 to 72 h. When required, antibiotics (purchased from Sigma Aldrich, MO, USA) were used to supplement the media and included erythromycin (10 μg/mL), thiamphenicol (10 μg/mL), and d-Cycloserine (250 μg/mL). All *E. coli* strains used (Table 1) were derivatives of HB101(pVS520) [27], Top 10 (Invitrogen, Carlsbad, CA, USA) or DH5α (Life Technologies, Carlsbad, CA, USA) and were grown aerobically in 2 × YT medium [28]. Where required, *E. coli* cultures were supplemented with appropriate antibiotics including chloramphenicol (25 μg/mL) or tetracycline (10 μg/mL).

### 2.2. Molecular and Genetic Techniques

Plasmid DNA was isolated from *E. coli* broth cultures, which had been grown overnight, using the QIAprep spin miniprep kit (Qiagen, Hilden, Germany) according to the manufacturer’s instructions. *C. sordellii* genomic DNA was prepared, as previously described [33]. All other molecular techniques were as previously described [34]. Nucleotide sequencing was performed by Micromon (Monash University, Melb, Australia) and was carried out using a Prism BigDye terminator cycle sequencing kit according to the manufacturer’s instructions (Applied Biosystems, MA, USA). Southern hybridization analysis was performed as previously described [8]. All plasmids used are shown in Table 1.

### 2.3. Construction of a nanS-Targeted TargeTron and a C. sordellii ATCC9714 nanS Mutant

Identification of high-scoring target insertion sites was determined as previously described [8]. The software “Intron Finder” (T. Seemann, personal communications) was used to identify potential *nanS* targetron insertion sites. Three oligonucleotide primers, DLP48 5′ CAGATTGTACAAATGTGGTGATAACAGATAAGTCGATTCAACTAACTTACCTTTCTTTGT 3′ (EBS1), DLP46 5′ TGAACGCAAGTTTCTAATTTCGATTATACGTCGATAGAGGAAAGTGTCT 3′ (EBS2) and DLP47 5′ AAAAAAGCTTATAATTATCCTTACGTATCGATTCAGTGCGCCCAGATAGGGTG 3′ (IBS), required for retargeting of the group II intron to the *nanS* target site were determined using this program. Inactivation of the *nanS* gene was performed as previously described for a similar inactivation of *tcsL* [8]. Here, splicing by overlapping extension PCR (SOE-PCR) was used to generate a 350 bp PCR product, which retargeted the intron to *nanS*. The PCR product was then cloned into the clostridial targetron plasmid pJIR3566 [32] using the *Bsr*GI/*Hin*dIII restriction enzyme sites. The resulting recombinant plasmid, designated pDLL35, was introduced into *E. coli* HB101 (pVS520) by electroporation to facilitate the conjugative transfer of pDLL35 into *C*. *sordellii* strain ATCC9714. This strain was passaged as previously described [8] to allow TargeTron insertion from pDLL35 into the chromosomal *nanS* gene, resulting in the construction of a strain ATCC9714 *nanS* mutant, designated DLL5034.

### 2.4. Complementation of the C. sordellii nanS Mutant

To construct a *nanS* complementation vector, the *nanS* gene, including 600 bp of upstream sequence to include relevant transcriptional signals such as a promoter, was PCR amplified using ATCC9714 genomic DNA with the oligonucleotide primers DLP202 5′ CGCGGATCCGGAGCCGCTGTTATCCC 3′ and DLP233 5′ CGAGCTCGGCAACTGGCTGGCATAG 3′ to produce a 1654 bp fragment. This fragment was successfully cloned into the *Sac*I/*Bam*HI sites of plasmid pRPF185, resulting in the construction of plasmid pDLL197 as confirmed by restriction enzyme analysis using *Sac*I/*Bam*HI and by nucleotide sequencing using the oligonucleotide primers DPL39 5′ TTCGTTATAATGGAGCAGCAGATCA 3′, DLP40 5′ GGAACTTTATTTCCCATTGTCCATG 3′, DLP202 and DLP233. Plasmid pDLL197 was introduced into HB101 (pVS520) by electroporation, resulting in the construction of strain DLL240. RP4-mediated conjugation was used to introduce pDLL197 into the *C. sordellii* strain ATCC9714 *nanS* mutant DLL5034, resulting in the isolation of DLL5101, the *nanS* complemented strain.

### 2.5. Sialidase Quantification Assays

**Figure 1:**
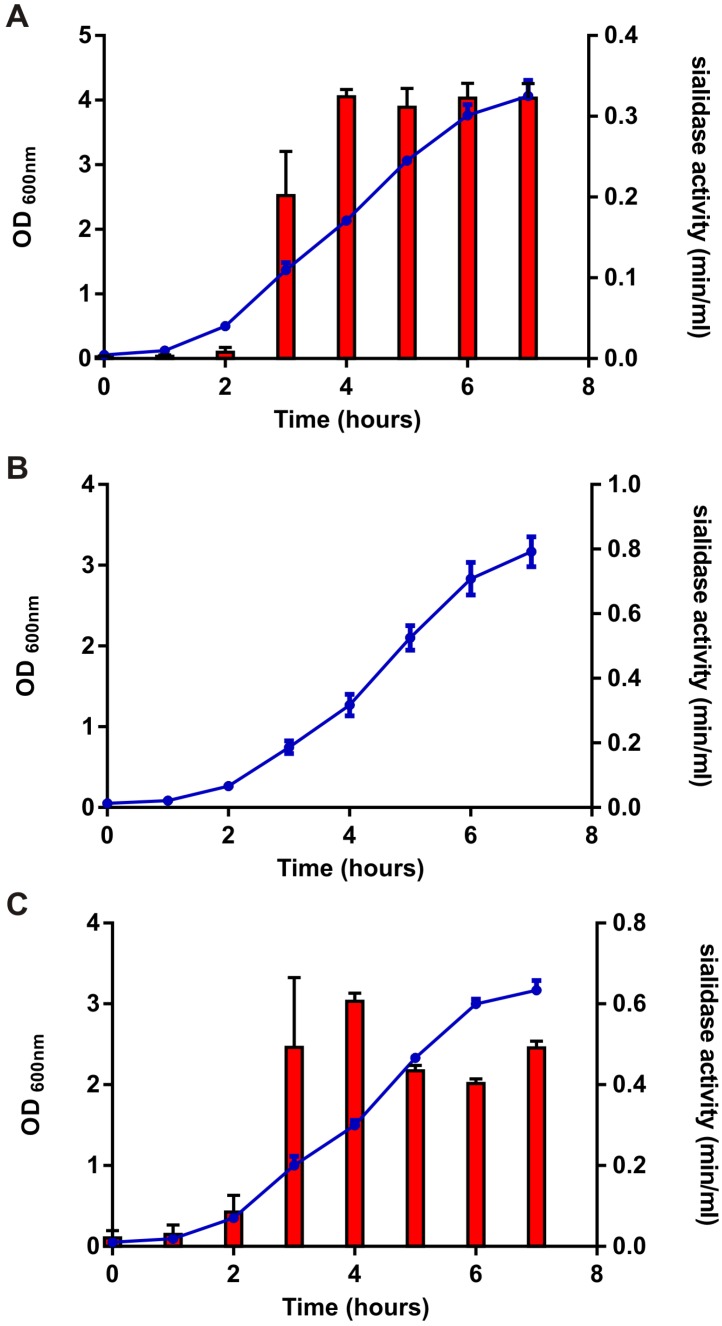
Growth rate analysis and sialidase activity from the *nanS* isogenic panel of strains. The wild-type (ATCC9714), *nanS* mutant (DLL5034) and *nanS* complemented strain (DLL5101) were grown for a maximum of 7 h in Todd Hewitt broth. Culture turbidity was measured hourly at OD_600 nm_. The results are shown on the left hand side of each graph (Figure 1**A**–**C**, *y*-axis; left hand side; blue lines). Sialidase activity (min/mL at pH 6) was also measured at hourly intervals and is shown on the right hand side of each graph (Figure 1**A**–**C**, *y*-axis; right hand side; red bars). All results represent the average of at least three independent experiments, each performed in duplicate. All error bars represent the standard error of the mean (SEM).

The presence of sialidase in culture supernatants was determined as previously described, with some modifications [17]. Anaerobic TH broths supplemented with glucose (0.02%), and cysteine 0.005%) were inoculated with an overnight culture (OD_600 nm_ = 0.05) of each strain and grown at 37 °C in an anaerobic chamber. 15 mL samples from each strain were taken at time zero and at hourly intervals up to 7 h post inoculation. The OD_600 nm_ was also determined at the time of sample collection. Culture supernatants were harvested by centrifugation at 5800× *g* for 10 min at 4 °C and frozen at −20 until required. To determine the optimal pH range for sialidase activity, the supernatant from the wild-type strain grown in TH for 4 h was tested using 0.5 M sodium acetate buffers ranging between pH 4.5 and pH 6.0. Under the conditions tested, the optimal pH for sialidase activity was determined to be 6.0 for the supernatant preparations (Figure S1). All subsequent experiments were therefore carried out using buffers at pH 6.0. To determine the optimal time after inoculation for sialidase production, all samples (taken hourly) were tested for the presence of sialidase using 4 mM of the substrate 5-bromo-4-chloro-3-indolyl-*D*-*N*-acetylneuraminic acid (Sigma) as previously described [17]. The optimal time for sialidase activity was determined to be 4 h post inoculation (Figure 1). Note that TcsL is not produced at a detectable level in this medium and at this time point. To determine the sialidase concentration in the culture supernatants, 10 μL of sodium acetate buffer (pH 6) was added to 20 μL of culture supernatant in a 96 well microtitre plate. Twenty microliters of the substrate was then added to each sample and the absorbance at 37 °C (620 nm) immediately determined at 2 min intervals for up to 120 min, using a TECAN 200 Infinite microplate reader. One unit of sialidase was defined as the change in absorbance/min at 620 nm/mL of sample. The culture supernatants were harvested at 4 h post inoculation. The data represents at least three independent experiments with each sample tested in duplicate.

### 2.6. Cell Cytotoxicity Assays

To determine the effect of sialidase with or without TcsL or other toxins on cell cytotoxicity the following cell lines were used: VK2/E6E7 (ATCC CRL-2616), a human vaginal (mucosal) epithelial cell line, End1/E6E7 (ATCC CRL-2615), a human endocervical epithelial cell line, Ect1/E6E7 (ATCC CRL-2614), a human ectocervical cell line, and Caco-2, (ATCC HTB-37,) a human colon carcinoma cell line. The first three of these were cultured in Complete Keratinocyte media (Life Technologies, CA, USA) supplemented with 0.4 mM CaCl_2_, 0.1 ng/mL Human recombinant EGF, 0.05 mg/mL Bovine pituitary extract, 50 μg/mL penicillin and 50 μg/mL streptomycin, and all were seeded at 2 × 10^5^ cells/mL in 96 well microtitre plates. Caco-2 cells were cultured in Dulbecco’s Modified Eagle’s Medium (DMEM) (Monash media preparation facility, Monash University, Clayton, Australia) supplemented with 10% heat-inactivated foetal calf serum (HI FCS), 100 units/mL penicillin and 100 μg/mL streptomycin, and seeded at 1.5 × 10^5^ cells/mL. All cell lines were incubated in an atmosphere containing 5% CO_2_ at 37 °C. Sialidase-containing supernatants were harvested at the optimal production time of 4 h post inoculation, as described above, and each cell line was exposed to twofold serial dilutions of culture filtrates diluted in the appropriate medium for 24 h at 37 °C. Media controls were also included and were carried out in duplicate. Changes in cell morphology were determined via microscopy (Olympus IX71 inverted microscope, Tokyo, Japan) and the endpoint scored visually as the last dilution at which 75%–100% cytopathic effects (CPE) were observed.

To determine the effect of sialidase on toxin-mediated cell cytotoxicity, including TcsL, two different culture supernatants were used in combination. Sialidase-containing supernatants were obtained as described earlier, using TH medium and a growth time of 4 h. Toxin-enriched supernatants, including TcsL, were obtained as described previously [8] with the following modifications. *C. sordellii* strain ATCC9714 or DLL5002 (*tcsL* mutant strain) were grown in 100 mL of TY broth for 72 h at 37 °C in an anaerobic chamber (Coy) and the supernatant harvested by centrifugation at 10,000× *g* for 8 min at 4 °C. The supernatant was then filtered (0.22 μm) (Sartorius Minisart syringe filter) and concentrated 20× using Vivaspin 20 100,000 MWCO PES spin concentrators (Sartorius). To perform the assays, the four eukaryotic cell lines described earlier were pre-treated with the undiluted sialidase-enriched supernatants from the wild-type strain ATCC9714, the *nanS* mutant DLL5034 or the *nanS* complemented strain DLL5101, for 3 h at 37 °C in an atmosphere containing 5% CO_2_, to allow the sialidase enzymes to act on the various cell lines. These supernatants were then removed and serial 2-fold dilutions of the toxin-containing supernatant or *tcsL*-mutant supernatant, diluted in the appropriate media, were added to each cell line or Ect1/E6E7 cells alone, respectively. Changes in cell morphology were determined *via* microscopy (Olympus IX71 inverted microscope) and the endpoint scored visually as the last dilution at which 75%–100% cytopathic effects (CPE) were observed. The data are presented as the average of five independent experiments, each with two replicate samples performed.

### 2.7. Cell Viability Assays

Cell viability assays were performed using the tissue culture plates described above to provide a quantitative measure of cell viability of toxin-treated cells that had been pre-treated with sialidase-containing culture supernatants. To perform these assays a Celltiter 96 cell Proliferation Assay (Promega, WI, USA) was used as per manufacturer’s instructions with slight modifications. The tetrazolium compound 3-(4,5-dimethylthiazol-2-yl)-5-(3-carboxymethopheny)-2-(4-sulfophenyl)-2H-tetrazolium inner salt, or MTS (Sigma) was prepared at 2 mg/mL in Dulbecco’s Phosphate Buffered Saline (2.7 mM KCl, 1.5 mM KH_2_PO_4_, 136.9 mM NaCl, 8.9 mM Na_2_HPO_4_.7H_2_O) (DPBS) and the electron coupling reagent (phenazine methosulfate) PMS (Sigma) was prepared at 0.92 mg/mL in DPBS. Before use, 100 μL of PMS was mixed with 2 mL of MTS and 20 μL of this solution was added to each well containing 100 μL of tissue culture medium. Plates were then read after a 1 h incubation at 37 °C in a 5% CO_2_ incubator, using a Tecan infinite M200 plate reader. This assay measures cell viability or activity since the absorbance at 490 nm is directly proportional to the number of living cells. The data is presented as the average of between three to five independent experiments, each performed in duplicate, and is plotted as a percentage of cell death *versus* toxin titre.

## 3. Results

### 3.1. Construction of a C. sordellii nanS Mutant

The *nanS* gene is present in all *C. sordellii* isolates [26] and NanS is considered to be a virulence factor of this bacterium, which may contribute to the characteristic leukemoid reaction that is associated with fatal cases of disease [5]. *C. sordellii* strain ATCC9714, which was isolated from an infected patient [30], encodes the *nanS* gene. Targetron technology was used in this study to construct a *C.* *sordellii* *nanS* mutant. Following the identification of a suitable *nanS* intron insertion site using Intron Finder, the targetron within the clostridial plasmid pJIR3566 [32] was retargeted to integrate at nucleotide position 304 on the direct DNA strand of the *nanS* gene, resulting in the construction of pDLL35. Using RP4-mediated conjugation, pDLL35 was introduced into *C. sordellii* strain ATCC9714, resulting in the isolation of thiamphenicol resistant transconjugants. Following the passage of this strain in erythromycin-containing media, erythromycin resistant, thiamphenicol sensitive putative *nanS* mutants were isolated. PCR and Southern blotting were then used to confirm the successful inactivation of the *nanS* gene.

The oligonucleotide primers DLP39 and DLP40 were used to generate the expected 400 bp PCR fragment from the wild-type strain; by comparison, a 2.2 kb fragment was obtained for the *nanS* mutant, as anticipated (data not shown). To further genetically confirm the *nanS* mutation in DLL5034, Southern blotting was performed using *Xba*I/*Xmn*I digested genomic DNA from the wild-type and mutant strain together with hybridization with a *nanS*-specific or an intron-specific probe. As expected, when probed with *nanS*, a 1.25 kb band was detected with the wild-type strain (Figure S2A) but no band was detected from the wild-type using an intron-specific probe (Figure S2B). The *nanS* mutant, DLL5034, showed the expected bands of 1.1 kb and 1.7 kb when probed with *nanS* (Figure S2A) and a single band of approximately 1.7 kb when probed with an intron-specific probe (Figure S2B). These results confirmed that a *C*. *sordellii* *nanS* mutant in strain ATCC9714, designated DLL5034, had successfully been constructed.

### 3.2. Complementation of the nanS Mutation

To construct a complete panel of isogenic strains appropriate for subsequent analysis, the *nanS* mutation in DLL5034 was complemented in *trans* with the wild-type *nanS* gene. The complementation vector, pDLL197, contains a chloramphenicol (*catP*) resistance cassette. First, plasmid pDLL197 was introduced into the *E. coli* strain HB101 (pVS520). This strain was then used as a donor in a conjugative mating to transfer pDLL197 into DLL5034, with chloramphenicol selection imposed in selective medium to ensure plasmid inheritance by the *C*. *sordellii* strain. *nanS*-specific PCR analysis using the oligonucleotide primers DLP39 and DLP40 confirmed the successful introduction of the complementation plasmid into DLL5034 through the generation of a 1800 bp DNA fragment specific to the complementation vector (data not shown).

#### 3.2.1. NanS is the Only Sialidase Produced by ATCC9714

Some clostridial species, such as *C*. *perfringens*, encode more than one sialidase [17]. The *C. sordellii* strain ATCC9714 appears to encode only one sialidase enzyme, encoded by the *nanS* gene. To confirm that the sialidase activity detected from ATCC9714 was the result of NanS alone, sialidase assays were performed using the isogenic panel of strains described above. The results showed that sialidase was secreted extracellularly and that maximal activity occurred from culture supernatants harvested at 4 h post inoculation (Figure 1). Sialidase could only be detected in culture supernatants harvested from the wild-type (0.32 units/min/mL, Figure 1A) and *nanS*-complemented strains (0.6 units/min/mL, Figure 1C). The increased sialidase activity detected from the complemented strain compared to the wild-type is likely to result from a dosage effect since *nanS* is present on a multi-copy plasmid in the complemented derivative. Sialidase activity could not be detected in culture filtrates isolated from the *nanS* mutant (Figure 1B). Bioinformatic analysis of NanS using Signal P [35] indicated that a signal peptide is present within this protein, with a cleavage site predicted to occur between amino acids 27 and 28. The psortb program [36] was also used to determine the potential localisation of the protein, which suggested that NanS is not cytoplasmically located and is likely to be secreted extracellularly, supporting our findings.

#### 3.2.2. NanS Is Not Cytotoxic to Eukaryotic Cells but Augments *C. sordellii* Cytotoxicity

Previous work has shown that sialidases can increase the cytotoxic effects of various toxins on eukaryotic cells [16,17]. To investigate the effect of NanS on *C*. *sordellii*-mediated cytotoxicity, we pre-incubated the vaginal (VK2), cervical (End1/E6E7 and Ect1/E6E7) and gastrointestinal (Caco2) cell lines for 3 h with the sialidase-enriched supernatants isolated from the isogenic panel of *nanS* strains. These supernatants were then removed from each well and toxin-containing supernatants (which contain TcsL) were added to the appropriate eukaryotic cells in a 2-fold dilution series. The resulting cytotoxic effects on each eukaryotic cell line were determined in two ways, qualitatively *via* visual examination and scoring and through the use of a quantitative MTS viability assay. For each test, five biological replicates were carried out in duplicate with appropriate statistical analysis applied, specifically, Mann-Whitney tests for the qualitative results and ANOVA (Kruskal Wallis) analysis for the quantitative results, with individual differences detected using Dunn’s multiple comparisons.

**Figure 2:**
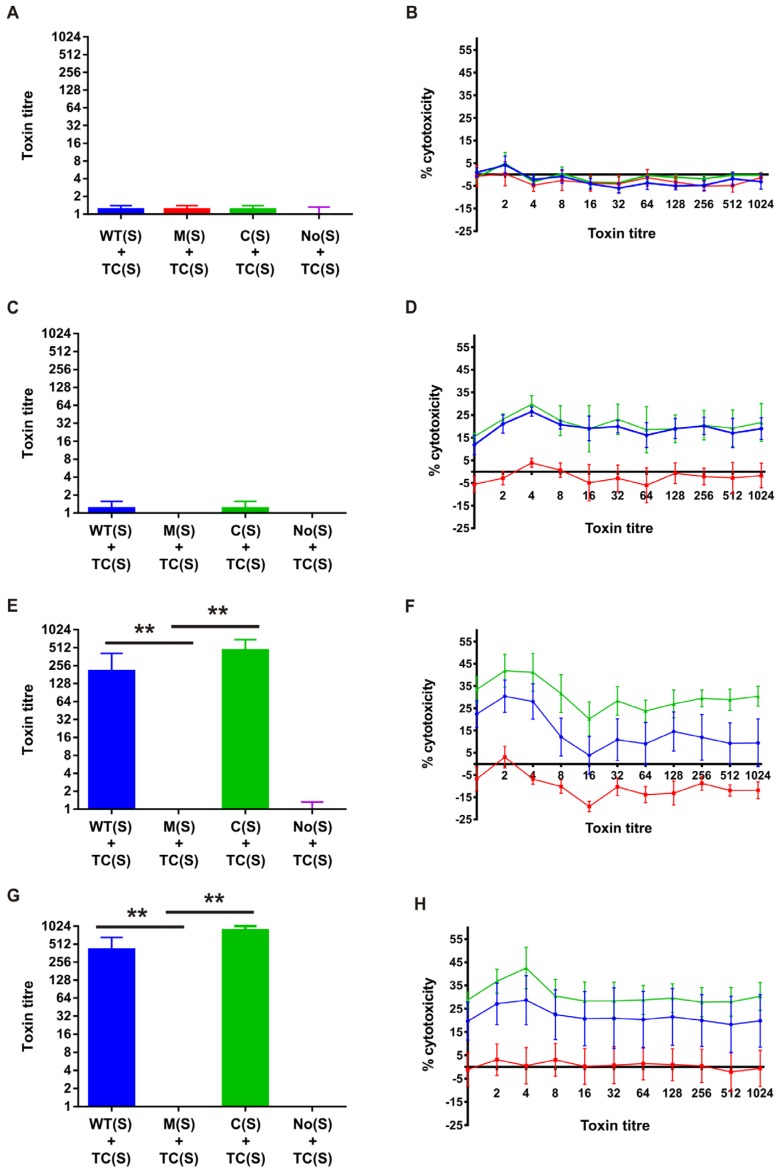
Cell cytotoxicity assays. Caco2 ((**A**) and (**B**)), VK2 ((**C**) and (**D**)), Ect1/E6E7 ((**E**) and (**F**)), and End1/E6E7 ((**G**) and (**H**)) cells were incubated for 3 h with *C. sordellii* sialidase-enriched supernatants, which were then removed by aspiration. Doubling dilutions of TcsL-enriched supernatants (containing other toxins) were then added to these cells and the mixtures incubated for 24 h. Morphological changes (cytopathic effects or CPE) were scored visually via microscopy (Figure 2A,C,E,G) and the last dilution at which CPE was observed was defined as the endpoint. Cell viability MTS assays were used as a quantitative measure of cytotoxicity (Figure 2B,D,F,H). The toxin titre is the reciprocal of the dilution endpoint and is shown for culture supernatants isolated from the wild-type (WT(S)) (blue), *nanS* mutant (M(S)) (red), *nanS* complemented (C(S)) (green) strain, or no supernatant (No(S)) control, and for the toxin-containing (TC(S)) supernatant. The data represents the average of five biological replicates, each performed in duplicate. The error bars in Figure 2A,C,F represent the standard error of the mean (SEM) as calculated by the Mann Whitney U test. The asterisks (**) represent a *p*-value of <0.005. The error bars in Figure 2B,D,F,H represent the SEM as calculated by ANOVA (Kruskall Wallis), with individual differences detected using Dunn’s multiple comparisons (VK2 cells: WT *versus* M, *p* < 0.05; M *versus* C, *p* < 0.001. Ect1/E6E7: WT *versus* M, *p* < 0.01; M *versus* C, *p* < 0.001. End1/E6E7: WT *versus* M, *p* < 0.01; M *versus* C, *p* < 0.001; WT *versus* C, *p* < 0.01).

Culture supernatants containing NanS were not cytotoxic to any of the cell lines used (data not shown). Similarly, very little cytotoxicity was detected in any of the cell lines when they were treated with the toxin-containing supernatants alone (Figure 2A,C,E,G). Pretreatment with NanS did not enhance cytotoxic activity on the gastrointestinal (Caco-2) cell line (Figure 2A) but slightly increased cytotoxic activity on the vaginal (VK2) cell line (Figure 2C). Strikingly, however, NanS significantly augmented the cytotoxic effects of these supernatant preparations on the cervical cell lines (End1/E6E7 and Ect1/E6E7, Figure 2E,G, respectively). In both the End1/E6E7 and Ect1/E6E7 cell lines, the cytotoxic effects of these toxin-containing supernatants on cells that had been pretreated with the sialidase-containing supernatants from the wild-type (blue lines) and *nanS*-complemented strains (green lines) were dramatic, with titres between 207 and 870 detected. By comparison, a toxin titre of less than 1 was detected when these cells had been pretreated with supernatant from the *nanS* mutant strain DLL5034 (red lines) (Figure 2E,G).

The results described above were supported by the quantitative data obtained through the use of the MTS cell viability assays. Caco2 cell viability was not altered in the presence of toxins present in the culture filtrate, including TcsL, even when cells had been pretreated with NanS-containing culture supernatants (Figure 2B). VK2 cell survival resulting from toxin-containing supernatants, however, was reduced when these cells were pretreated with NanS. In this cell line, 19.2% and 21.2% toxin-mediated cytotoxicity was observed for cells pretreated with the NanS-containing supernatants from the wild-type and complemented strains, respectively. Toxin-mediated cytotoxicity was, however, abolished (<1%) when the cells were pretreated with supernatants from the *nanS* mutant strain.

The results obtained above for the End1/E6E7 and Ect1/E6E7 cell lines were also supported by the MTS cell viability assays. In the Ect1/E6E7 cells, 15% toxin-mediated cytotoxicity was detected when cells had been pretreated with wild-type NanS-containing culture supernatant whereas less than 1% cytotoxicity was detected when cells had been pretreated with *nanS* mutant supernatant (Figure 2F). Cytotoxicity levels were restored when cells were pretreated with culture supernatant from the complemented strain, with 30.5% cytotoxicity detected (Figure 2F). The increase in activity from the complemented strain is likely to result from the greater level of sialidase production that occurs from this strain because of the multi-copy nature of the *nanS*-carrying plasmid, pDLL197 (Figure 1C). Similarly, 22% toxin-mediated cytotoxicity was detected from End1/E6E7 pre-exposed to the NanS-containing supernatant from the wild-type strain (Figure 2H). These levels decreased dramatically in the absence of NanS pretreatment (<1%) but rose upon *nanS*-complementation (30.8%) to levels above those detected when supernatants from the wild-type strain were applied prior to exposure to toxin-containing supernatants (Figure 2H).

#### 3.2.3. *C. sordellii* Cytotoxicity Enhanced by NanS Is Not TcsL-Mediated and Results from the Action of an Unknown Toxin or Toxins

**Figure 3:**
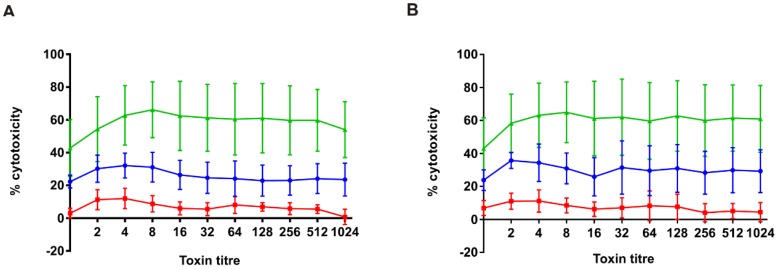
Cell cytotoxicity assays. Ect1/E6E7 cells were incubated for 3 h with *C. sordellii* sialidase-enriched supernatants, which were then removed by aspiration. Doubling dilutions of supernatants from either the TcsL-containing wild-type strain (ATCC9714) (**A**) or the *tcsL* mutant strain DLL5002 (**B**) were then added to these cells and the mixtures incubated for 24 h. Cell viability MTS assays were used as a quantitative measure of cytotoxicity. The toxin titre is the reciprocal of the dilution endpoint and is shown for culture supernatants isolated from the wild-type (WT(S) (blue)), *nanS* mutant (M(S) (red)), and the *nanS* complemented (C(S) (green)) strain. The data represents the average of three biological replicates, each performed in duplicate. The error bars represent the SEM as calculated by ANOVA (Kruskall Wallis), with individual differences detected using Dunn’s multiple comparisons (Ect1/E6E7: WT *versus* M, *p* < 0.01; M *versus* C, *p* < 0.001).

The semi-purified toxin-containing supernatants described above contain TcsL in addition to numerous other unidentified proteins. To determine if NanS was specifically enhancing the cytotoxic effects of TcsL, the cytotoxicity assays described above were performed using supernatants harvested from a previously constructed *tcsL* mutant, DLL5002. These supernatants were applied to Ect1/E6E7 cells following exposure of the cells to sialidase-enriched supernatants from either the wild-type, *nanS* mutant or *nanS* complemented strains, as before. Unexpectedly, the cytotoxic effects of these culture supernatants were comparable, with no difference in cytotoxicity observed from TcsL-containing supernatants (Figure 3A) compared to those without this toxin (Figure 3B). In the Ect1/E6E7 cells, 26% and 30% toxin-mediated cytotoxicity was detected upon exposure of these cells to the TcsL-containing (TcsL^+^) supernatant or the *tcsL* mutant strain (TcsL^−^) supernatant, respectively, following pretreatment with wild-type NanS-containing supernatant (blue lines). By contrast, only 7% cytotoxicity was detected when cells had been pretreated with the *nanS* mutant strain supernatant and then treated with the TcsL^+^ and TcsL^−^ culture supernatants (Figure 3A,B; red lines). Cytotoxicity levels were restored when cells were pretreated with culture supernatant from the *nanS* complemented strain, with an average of 59% and 60% cytotoxicity detected in the presence of the supernatants derived from the TcsL^+^ and TcsL^−^ supernatants, respectively (Figure 3A,B; green lines). These results suggest that TcsL is not responsible for the enhanced cytotoxicity observed with Ect1/E6E7 cells in the presence of NanS but that another, unidentified toxin or toxins is responsible for this phenotype.

## 4. Discussion

*C. sordellii* causes severe infections in humans and animals [37] and is associated with significant mortality rates [5]. Although *C. sordellii* has been implicated in intestinal diseases of animals [5,38,39], soft tissue and intrauterine infections appear to be more common in humans [2,5]. The distinguishing features of *C. sordellii* infections in human clinical cases include profound capillary leak syndrome and a leukemoid reaction that generally exceeds 100,000 white blood cells per μL of blood [1]. The sialidase, NanS was previously purified and shown to induce the proliferation of promyelocytic cells *in vitro*, suggesting that it may contribute to the leukemoid reaction [1]. In this study we sought to examine the role of NanS in the pathogenesis of *C. sordelli*-mediated disease. Insertional inactivation of the *nanS* gene followed by assays for sialidase production showed that this phenotype was abolished under the conditions tested but was restored in the mutant strain complemented with the wild-type *nanS* gene. This work suggests that *C. sordellii* strain ATCC9714 only produces one sialidase, NanS, a finding which may apply to most other isolates since all *C. sordellii* strains assessed to date appear to encode only one sialidase gene, *nanS* [1,7,26]. NanS has a predicted signal peptide and is secreted from the cell into the culture supernatant, similar to NanI from *C.* *perfringens* [17]. While *C. perfringens* produces three sialidases (NanI, NanJ and NanH), NanI is the major secreted sialidase and contributes to the increased cytotoxicity of a number of *C*. *perfringens* toxins when eukaryotic cells are pretreated with this enzyme [16,21,24].

Sialic acids are found ubiquitously within the host and contribute to the promotion of important cell-to-cell interactions, stabilizing the conformation of glycoproteins and concealing ligand binding receptors [10]. Sialylated mucus-associated glycoproteins, including MUCs (mucin proteins), IgA and lactoferrin, play important roles in protecting the host from invading microorganisms by providing a physical barrier, lubricating epithelial surfaces and exhibiting bactericidal properties [15]. Bacteria produce sialidases which act on host sialic acids and which have numerous beneficial roles for the bacteria that produce them including the provision of nutrients, facilitating colonisation, promoting biofilm production and overcoming the host immune response [15]. Sialidases also enhance the cytotoxicity of major bacterial toxins encoded by pathogens, including those produced by *C. perfringens* and *V. cholera* [16,17,22]. Here, we showed that NanS was not directly cytotoxic to gastrointestinal, vaginal or cervical cell lines. However, the cytotoxic effects of a toxin-containing supernatant were augmented when vaginal or cervical cells were pretreated with the NanS sialidase.

These results mirror those obtained with NanI from *C. perfringens*. In *C. perfringens*, NanI was not directly cytotoxic to eukaryotic cells but enhanced the cytotoxic effects of the epsilon toxin (ETX), a toxin responsible for intestinal diseases in animals [16,21]. The removal of sialic acid residues from the surface of eukaryotic cells by NanI is thought to expose additional epsilon toxin (ETX) receptor sites leading to increased binding and ETX complex formation and resulting in enhanced ETX-mediated cytotoxicity [16]. In *V. cholera*, the sialidase NANase is thought to increase the local concentration of the cholera toxin (CT) receptor, GM_1_, on the cell surface which serves to increase binding and internalisation of CT [22]. In the same way, the NanS-mediated enhancement of cytotoxicity seen with the reproductive cell lines here may result from NanS cleavage of sialic acid residues from mucus related sialoglycans. Cleavage of these sialic acid residues would render these mucins susceptible to further degradation by glycoside hydrolases [15] thereby unmasking or increasing the concentration of toxin-specific receptors on the epithelial surface, resulting in increased toxin uptake and higher levels of cellular destruction. Moreover, contact of *C. sordellii* with cells from the reproductive tract might result in an increase in the local concentration of NanS which would serve to enhance toxin-mediated cytotoxicity. This phenotype was observed in studies on NanI where production of this enzyme was augmented when *C. perfringens* cells came into contact with intestinal cells [16].

In contrast to the reproductive cell lines used in this study, the Caco-2 intestinal cell line appeared to be unaffected by the toxin-containing supernatants and this effect was not altered when the cells were exposed to NanS. It is known that different mucosal sites secrete sialoglycans that are structurally distinct [15] so it is possible that NanS has a higher affinity for sialoglycans of the reproductive tract and a lower affinity to those found on intestinal cells although additional intestinal cell lines would need to be investigated to test this hypothesis. Alternatively, it is possible that Caco-2 cells lack the receptor(s) required for toxin binding and that these cells are not susceptible to intoxication by *C*. *sordellii* toxins.

As well as the enhancement of cytotoxicity, NanS may be playing a role in *C*. *sordellii* infections in a broader context. Some bacterial sialidases, such as SabA from *Helicobacter pylori* [40], increase bacterial adherence to host cells. Other microbial sialidases serve to subvert the host immune response. For example, the deglycosylation of human serum glycoproteins by three exoglycosidases encoded by *Streptococcus pneumoniae*, including the major sialidase NanA, promoted resistance to bacterial complement-dependent killing and phagocytosis by human neutrophils [41]. *Trypanosoma cruzi* escapes the immune response by using its trans-sialidase enzyme to transfer sialic acid residues from host glycoproteins to acceptor molecules found on the parasite cell surface [42]. Similarly, *Neisseria meningitidis* and *Escherichia coli* K1 produce sialidases that act to decorate their surfaces with sialic acid residues to aid their escape from the host immune response [43]. The involvement of NanS in adherence or immune evasion during *C*. *sordellii* infections, however, remains to be determined.

The microbiota of the gut and reproductive tract vary considerably. The gut is colonized predominantly by *Bacteroides* species and Firmicutes [44] while the vagina is colonized predominantly by *Lactobacillus* species [45]. By comparison, the cervical microbiota has not been well defined [46,47]. Gut commensal bacteria, including various *Bacteroides* and *Clostridium* species, express sialidases [48], some of which have been utilized by pathogens to provide a fitness advantage. The gut commensal *Bacteroides thetaiotaomicron*, for example, produces a sialidase that cleaves sialic acids from intestinal mucins which provides a nutritional advantage for the proliferation of gut pathogens such as *Salmonella typhimurium* and *Clostridium difficile* [49,50]. Although less well described, sialidase producers in the reproductive tract have been identified, including *Prevotella* species and *Bacteroides fragilis* [51]. Of interest, however, was the observation that sialidase was absent in the vagina of healthy women but was detected in women with bacterial vaginosis [15]. *C. sordellii* has been found in female reproductive tissues, including the vagina, and induces inflammation in cervical tissues and also causes intrauterine infections [5,52]. It is possible that sialidases produced by commensal bacteria may play a role in enhancing *C*. *sordellii* cytotoxicity or may act to provide additional nutrients that may assist in *C*. *sordellii* growth or colonisation, and that this activity is therefore not dependent on NanS alone, however, further studies are required to test this hypothesis.

Although toxin-mediated cytotoxicity in the vaginal cell line following NanS treatment appeared to be low when cells were visualised microscopically, results from the more sensitive cell viability assay suggested that similar levels of augmentation occurred with this cell line and with both cervical cell lines. Taken together, these results suggest that NanS may act in tissues from the female reproductive tract to enhance the effects of toxins secreted by *C*. *sordellii*, which is supported by the observation that *C. sordellii* has been found in female reproductive tissues. The rarity of infections [5,52] and low carriage rates [25] of *C. sordellii* in women suggest that this organism may be transiently located in this niche and may not be well suited for growth in the reproductive tract environment. However, NanS may play a role here in promoting the survival of *C. sordellii*, perhaps in a similar way to *S. pneumonia* and *Pseudomonas aeruginosa* through the promotion of biofilm formation [41,53] or through the provision of nutrients to facilitate growth and persistence. Successful colonisation may lead to a progression of infection throughout the reproductive tract, particularly in regions where the pH is optimal for NanS activity.

Clearly, NanS plays a role in augmenting the cytotoxic capacity of *C*. *sordellii*. NanS activity was found to be highest in a buffering environment of pH 6.0, a pH close to that found in the human female cervix [54]. It is therefore tempting to speculate that NanS would be optimally active in this anatomical site. Indeed, *C*. *sordellii* infections occur in the cervix, with acute inflammation and necrosis detected via cervical swabbing [52]. Infection of the cervix may enable deeper infections of the female reproductive tract including the uterus, an identified *C*. *sordellii* infection site in postnatal and post-abortive women [55,56]. The major toxin of *C*. *sordellii*, TcsL, appears to be optimally active at lower pH levels of between 4 and 5 [57,58]. The optimal pH conditions for NanS and TcsL therefore appear to be different although both are likely to be active during infections. However, the study presented here unexpectedly suggests that TcsL is not active against the reproductive cell line Ect1/E6E7 since the use of a *tcsL* mutant generated the same cytotoxicity levels as those of a TcsL-producing wild-type strain. Instead, this data suggests that an unidentified toxin or toxins is responsible for this cytotoxic activity, and that this activity is enhanced by the presence of NanS. NanS may therefore serve to exacerbate *C. sordelli*-mediated tissue damage and disease in the host, particularly in the female reproductive tract. The future identification and characterisation of this toxin(s) is important for our understanding of *C*. *sordellii* infections and disease, particularly in the context of tissue specificity of different cytotoxic factors.

## Figures and Tables

**Table 1:** Bacterial strains and plasmids used in this study.

Strain or Plasmid	Characteristics	Reference
**Strains**		
*E. coli*		
DH5α	F^−^ ϕ80d*lacZ*ΔM15 Δ(*lacZYA argF*)*U169 endA1 recA1 hsdR17* (r_K_^−^ m_K_^−^) *deoR thi-1 supE44 gyrA96 relA1*	Life Technologies
One-Shot TOP10	F^−^ *mcr*A (*mrr-hsdRMS-mcrBC*) 80*lacZ* M15 *lacX74 nupG recA1 araD139* (*araleu*) *7697 galU galK rpsL*(Str^r^) *endA1* λ ^_^	Invitrogen
HB101	*thi-1 hsdS20*(r_B_^−^ m_B_^−^) *supE44 recAB ara-14 leuB5 proA2 lacY1 galK rpsL20* (Sm^r^) *xyl-5 mtl-1*	[29]
*C. sordellii*		
ATCC 9714	Type strain; TcsL^+^ TcsH^−^ Nan^+^ SDL^−^ PLC^+^	[30]
DLL5034	ATCC 9714 *nanS*Ωtargetron; Em^r^	This study
DLL5101	DLL5034(pDLL197)	This study
DLL5002	ATCC 9714 *tcsL*Ωtargetron; Em^r^	[8]
**Plasmids**		
pVS520	Tra^+^ Mob^+^ RP4 derivative; Tc^r^	[27]
pRPF185	*E*. *coli*–*C*. *difficile* shuttle vector, *P*_tet_::*gusA cat CD6ori RP4oriT-traJ pMB1ori*	[31]
pJIR3566	Clostridial mobilizable targetron vector; with pCB102 replication region, *lacZ*, *oriT*; Cm^r^	[32]
pJIR3566	Group II intron of pJIR3566 retargeted to the 304/305s site of the *nanS* gene	This study
pDLL197	pRPF185 (*Sac*I/*Bam*HI) DLP202/DLP233 *nanS* PCR product (*Sac*I/*Bam*HI; 1654 bp); Cm^r^	This study

## Supplementary Materials

The following are available online at www.mdpi.com/2072-6651/8/6/189/s1, Figure S1: NanS activity under different pH conditions, Figure S2: Southern hybridization analysis of the *nanS* mutant.
