# Supplementary material for: The Sialidase NanS Enhances Non-TcsL Mediated Cytotoxicity of Clostridium sordellii
Source: Toxins (Basel). 2016 Jun 17;8(6):189. doi: 10.3390/toxins8060189 (PMC4926155; doi:10.3390/toxins8060189)
Supplement: Supplementary file 1 [file toxins-08-00189-s001.pdf]

Supplementary Materials: The Sialidase NanS Enhances Non-TcsL Mediated Cytotoxicity of *Clostridium sordellii*

Milena M. Awad, Julie Singleton and Dena Lyras

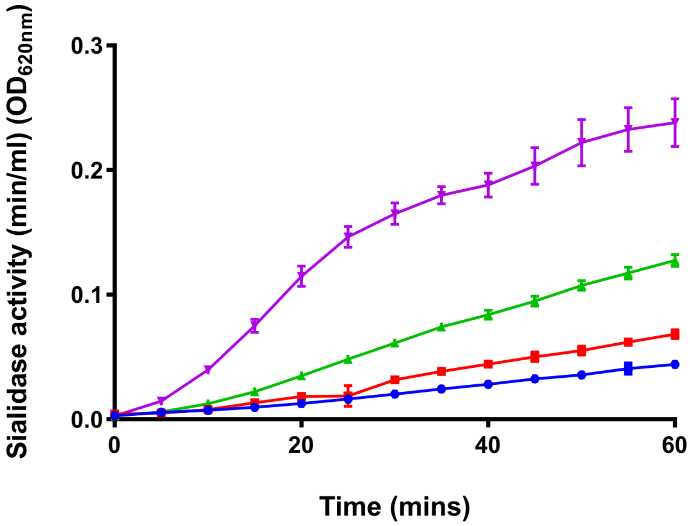

Figure S1. NanS activity under different pH conditions. Sialidase activity was determined in the culture supernatant isolated from the wild-type (ATCC9714) strain grown in Todd Hewitt (TH) broth for 4 h and was tested in acetate buffers ranging in pH from 4.5 to 6.0 over 60 mins. The pH range shown includes 4.5 (blue), 5.0 (red), 5.5 (green) and 6.0 (purple). The results are the average of three independent experiments, each performed in duplicate. The error bars represent the standard error of the mean.

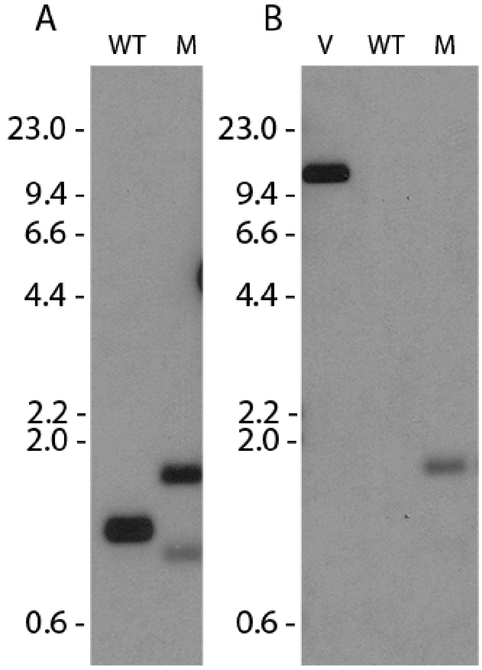

Figure S2. Southern hybridization analysis of the *nanS* mutant. Genomic DNA from the wild-type (ATCC9714) (WT) and *nanS* mutant (DLL5034) (M) was digested with *Xba*I/*Xmn*I, separated by electrophoresis using 0.8% agarose gels and transferred onto nitrocellulose. Hybridization was then performed with (**A**) a *nanS*-specific probe or (**B**) an intron specific probe, showing fragments of the expected size for both the wild-type and mutant strains.
